# Soil viruses drive carbon turnover during subtropical secondary forest succession

**DOI:** 10.3389/fmicb.2025.1633379

**Published:** 2025-09-19

**Authors:** Xingyi Chen, Danting Yu, Yuting Yan, Chengyu Yuan, Jizheng He

**Affiliations:** ^1^College of Geographic Sciences, Fujian Normal University, Fuzhou, China; ^2^Fujian Provincial Key Laboratory for Subtropical Resources and Environment, Fujian Normal University, Fuzhou, China; ^3^School of Agriculture, Food and Ecosystem Sciences, Faculty of Science, The University of Melbourne, Melbourne, VIC, Australia

**Keywords:** metavirome, auxiliary carbohydrate-active enzyme genes, carbon cycling, succession, secondary forest

## Abstract

**Introduction:**

Soil viruses are increasingly recognized as key regulators of microbial ecology and ecosystem function, yet their roles in forest ecosystems, particularly during natural secondary succession, remain largely unexplored.

**Methods:**

We examined soil viral communities across five successional stages of secondary forests to investigate their taxonomic dynamics and functional potential. Using high-throughput viral metagenomics, we characterized viral community structure, abundance, and auxiliary metabolic gene content.

**Results:**

Our results demonstrate that soil viral abundance and community composition shift significantly with forest stand age. Viral richness increased during succession, with compositional transitions observed across stages; however, tailed bacteriophages consistently dominated. Structural equation modeling and linear mixed-effects analysis identified soil pH and bacterial diversity as primary environmental determinants of viral diversity. Functionally, soil viruses harbored auxiliary metabolic genes related to carbohydrate metabolism, indicating their potential involvement in modulating host metabolic processes. Successional trends in viral functional profiles revealed a transition from carbon assimilation to carbon release pathways, suggesting viral mediation of carbon turnover. Notably, the enrichment of glycoside hydrolase and glycosyl transferase genes across forest ages implies a role for viruses in shaping microbial carbon processing capacities through carbohydrate-active enzyme contributions.

**Discussion:**

These findings provide novel evidence that soil viruses actively participate in ecosystem succession by influencing microbial functional potential and biogeochemical cycling. This study underscores the ecological importance of soil viral communities in regulating carbon dynamics during secondary forest development.

## 1 Introduction

Forest ecosystems are the largest carbon reservoir and the most efficient carbon sink in global terrestrial ecosystems, with approximately 45% of the world’s soil carbon stored in forest soils (Pan et al., 2011; Shi et al., 2024). Forest soils are crucial habitats for microorganisms and essential sites for the cycling of carbon, playing a significant role in enhancing the overall carbon sequestration function of forests, maintaining global carbon balance, and regulating global climate (Dixon et al., 1994). Studies have shown that soil microbial communities play a key role in nutrient cycling, plant growth, community dynamics, and ecosystem multifunctionality (Wardle et al., 2004; Bardgett and van der Putten, 2014; Delgado-Baquerizo et al., 2016). Moreover, key microorganisms can contribute significantly to climate change feedbacks through soil carbon cycling. Subtropical forests, with their unique geographical conditions and climate characteristics in subtropical regions (Li et al., 2022), are a major hotspot for global biodiversity and host a wealth of microbial resources. Their energy flow and material cycling are of great importance to the global biogeochemical cycles of elements (Sayer et al., 2020). Viruses, as an important component of soil microbial communities, are abundant but less studied compared to bacteria and fungi. Their impact on the carbon cycle of subtropical forest ecosystems remains poorly understood.

Viruses are the most abundant biological entities on Earth (Breitbart and Rohwer, 2005) and are key drivers of global biogeochemical cycles. They also represent the largest genetic reservoir on Earth (Suttle, 2005). In soil ecosystems, viruses play pivotal ecological roles, centrally manifested through two core functions: regulation of host population dynamics and propulsion of biogeochemical cycling (Huang et al., 2024). The specific mechanism is that viruses, on one hand, can serve as a medium for microbial evolution by driving host evolution through horizontal gene transfer, affecting the structure and function of microbial communities (Zablocki et al., 2016). On the other hand, they can acquire auxiliary metabolic genes (AMGs) from hosts and express them, thereby participating in the host’s metabolic and life processes, including photosynthesis (Thompson et al., 2011), carbon, nitrogen, and sulfur metabolism (Sullivan et al., 2010; Roux et al., 2016; Jin et al., 2019; Kieft et al., 2021), nucleic acid synthesis and metabolism (Enav et al., 2014), and phosphate metabolism (Kathuria and Martiny, 2011; Han et al., 2022), altering the productivity of ecosystems, and impacting biogeochemical cycles (Roux et al., 2016; Breitbart et al., 2018; Tian et al., 2024). It is evident that the long-term coexistence and interactions between soil viruses and host microorganisms are crucial for maintaining the ecosystem services of forest ecosystems.

In recent years, with the growing popularity of soil virus research, their impact on the carbon cycle has begun to attract widespread attention. Studies have shown that viruses influence elemental cycling primarily through bottom-up regulation, directly controlling elemental cycles by encoding auxiliary metabolic genes, and downward regulation by infecting and lysing host microorganisms, thus indirectly affecting the biogeochemical cycling of elements (Suttle, 2007; Jover et al., 2014; Emerson et al., 2018). The viral shuttle mechanism suggests that soil viruses can increase the abundance of refractory dissolved organic matter (DOM), which has relatively higher bioavailability to microorganisms and serves as an important substrate for microbial metabolism. Consequently, microbial carbon converted into recalcitrant DOM components through the biological pump mechanism may be an important soil carbon sink (Zimmerman et al., 2020; Chen et al., 2021; Tong et al., 2023). Additionally, the “viral shunt” model indicates that the substances released upon viral lysis of host cells can provide substrates for the growth of other microbial populations in the soil habitat. Therefore, viruses contribute to the soil organic carbon pool by transferring carbon from the host through lysis, thus enhancing the soil carbon sink (Shiah et al., 2022). Research has also shown that soil virus-induced lysis significantly increases the content of soluble carbon, promotes organic carbon turnover, and regulates CO_2_ emissions. At the same time, viral infection increases the accumulation of microbial biomass, improves microbial carbon use efficiency, accelerates microbial community turnover, and thus enhances the carbon sink function of forest soils (Zhou et al., 2024). In natural forest soils, bacteriophages can reduce the temperature sensitivity of CO_2_ in response to temperature rise, thereby mitigating the impact of global warming on organic carbon mineralization (Nicolas et al., 2023). The soluble organic matter derived from viral lysis can be converted into recalcitrant soluble organic matter and accumulate in the soil, promoting carbon sequestration in the soil (Tong et al., 2023). These findings highlight that soil viruses play a key role in the carbon cycle through various complex ecological mechanisms.

As the core of forest resources, the protection of natural forests is crucial for enhancing carbon sink functions and maintaining ecological balance. In addition to being influenced by factors such as forest age and tree species composition (Fanin et al., 2025; Lin et al., 2025), forest carbon sinks are also closely related to habitat and climate conditions (Cook-Patton et al., 2020). It is generally believed that fast-growing young and middle-aged forests have stronger carbon sequestration capabilities, while the carbon sink potential of old-growth forests and degraded forests is relatively low (Yu et al., 2014). However, some studies suggest that old-growth forests may also have strong carbon sink functions, particularly in terms of their ability to retain soil carbon (Zhou et al., 2006). Building on these perspectives, this study focuses on subtropical secondary forests of varying successional stages (8, 20, 27, 40, 100 years old) in the Baisha State-owned Forest Farm, aiming to elucidate the role of soil viruses in forest development and carbon cycling. Using viral metagenomic sequencing and advanced bioinformatics, we investigate: (i) the abundance and environmental drivers of soil viral and microbial communities across successional stages; (ii) the compositional and ecological characteristics of soil viral communities and their environmental determinants; (iii) the functional potential of soil viruses and their ecological implications for carbon cycling during forest succession.

## 2 Materials and methods

### 2.1 Study area overview and sample collection

The study area is located in the Baisha State-Owned Forest Farm in Shanghang County, Longyan City, Fujian Province (24 °46′ ~ 25 °28’ N, 116 °16′ ~ 116 °57′E), which belongs to the subtropical monsoon climate zone. The average annual temperature is 20.1 °C, and the annual precipitation is 1,600 mm (Wang et al., 2023). The forest farm is home to both artificial *Cunninghamia lanceolata* plantations and natural forests, with long-term experimental plots established. After disturbance, the natural forests have undergone natural restoration, resulting in secondary forests dominated by *Castanopsis carlesii*, *Castanopsis fargesii*, and *Castanopsis fissa* (Su et al., 2021).

In June 2018, soil samples were collected from the secondary forest of the region. To ensure a wide range of forest stand ages, five age gradients were set, namely 8 years (young forest), 20 years (middle-aged forest), 27 years (near-mature forest), 40 years (mature forest), and 100 years (over-mature forest). Three replicates were taken from each gradient, resulting in a total of 15 soil samples. Three sampling points were selected for each sample, a minimum separation distance of 3 meters was maintained between all sampling points, and surface soil (0–10 cm) was collected at each point using the five-point sampling method, then pooled together to form the final soil sample. The samples were immediately transported to the laboratory using ice boxes. All soil samples were passed through a 2 mm sieve to remove sand and roots, thoroughly mixed, and then divided into portions for storage at 4°C and −80°C for subsequent experiments.

### 2.2 Soil physicochemical properties measurement

Soil physicochemical properties were measured according to the methods outlined in the Soil Agrochemical Analysis (Bao, 2000), including ammonium nitrogen (NH_4_^+^-N), nitrate nitrogen (NO_3_^−^-N), dissolved organic carbon (DOC), dissolved organic nitrogen (DON), total carbon (TC), total nitrogen (TN), available phosphorus (AP), electrical conductivity (EC), soil water content (SWC), and soil pH.

NH_4_^+^-N and NO_3_^−^-N were extracted using 1 mol/L KCl solution, and measured using a continuous flow analyzer (Skalar san++, Holland). Dissolved organic carbon and organic nitrogen contents were extracted using 0.5 mol/L K_2_SO4 and measured using an organic carbon analyzer (TOC-VCPH, Shimadzu, Kyoto, Tokyo). TN and TC content in the soil was determined using air-dried soil passed through a 100-mesh sieve and measured using an elemental analyzer (Vario MAX cube, Elementar, Germany). AP in the soil was measured using the Olsen method with a continuous flow analyzer (SAN++, Skalar, Holland). EC was measured with a portable conductivity meter (S7-Standard Kit, Mettler Toledo, Germany) at a soil-to-water ratio of 1:5. SWC was determined by drying fresh soil in an oven at 105 °C until it reached a constant weight. Soil pH was measured using a pH meter (FE20-FiveEasy, Mettler Toledo, Germany) at a soil-to-water ratio of 1:2.5.

### 2.3 Fluorescent microscopic quantification of soil viruses and microorganisms

This experiment followed the protocols of Trubl et al. (2016) and Xiao et al. (2014) with modifications tailored to site-specific soil conditions. Key steps are summarized as follows: 3 g of fresh soil was weighed and mixed with 10 mL AKC buffer solution (1% potassium citrate + 10% PBS + 150 mM MgSO_4_), then shaken and incubated for 15 min. After incubation, the mixture was left to settle and the supernatant was collected. This extraction procedure was repeated three times. The collected extract was filtered through a 0.45 μm filter, and then passed through a vacuum filtration system to load it onto a 0.02 μm Anodisc Al_2_O_3_ filter membrane. After the filter membrane was dried in the dark, 100 μL of a 1:400 SYBR Green I solution (Invitrogen, Eugene, Oregon, United States) was added for staining. After 20 min, the filter was removed, dried, and then mounted with an anti-fade solution before being examined under a fluorescence microscope. Small and uniformly bright spots in the field of view were identified as virus particles, while larger, brighter spots were considered microorganisms. Three replicates were measured for each soil sample.

### 2.4 Total DNA extraction and amplicon sequencing

Total microbial DNA was extracted from soil samples using the FastDNA Spin Kit for Soil (MP Biomedicals, CA, United States) according to the manufacturer’s instructions. DNA quality and concentration were assessed, and qualified DNA samples were used for PCR amplification of the V4 region of the 16S rRNA gene with primers 515F/806R on an ABI GeneAmp PCR System 9700 (Thermo Fisher Scientific, United States). PCR products were verified by 2% agarose gel electrophoresis, and target bands were excised and purified using the AxyPrep DNA Gel Extraction Kit (AXYGEN, United States). Sequencing libraries were constructed using the TruSeq DNA Sample Prep Kit (Illumina, United States) and sequenced on the Illumina NovaSeq 6000 platform. Sequencing data were processed as follows: paired-end reads were demultiplexed based on sample-specific barcodes and subjected to quality control and filtering. High-quality reads were merged based on overlapping regions to generate optimized sequences. The DADA2 pipeline was then used for sequence denoising, yielding amplicon sequence variants (ASVs) and their corresponding abundance tables for downstream bioinformatic analysis.

### 2.5 Viral DNA extraction and metagenomic sequencing

The viral DNA extraction process was adapted from the method used by Han et al. (2017) for agricultural soils, with appropriate modifications: 50 g of fresh soil was weighed and mixed with 150 mL AKC buffer, then shaken and incubated for 15 min. The mixture was then centrifuged, and the supernatant was collected. This extraction step was repeated three times. Next, the supernatant was filtered using a tangential flow filtration system (Tangential Flow Filter System, QuixStand, GE Healthcare Life Sciences, Pittsburgh, PA, United States), sequentially through 0.6 μm, 0.45 μm, and 0.22 μm hollow fiber filter columns and a 30 kDa ultrafiltration column, concentrating the final filtrate to less than 100 mL. The concentrated solution was further filtered through a 0.22 μm sterile filter to remove any potential contaminants. Finally, the filtrate was concentrated using a 30 kDa ultrafiltration tube (Merck Millipore Ltd., Tμllagreen, IRL) until the volume was approximately 1 mL. Viral DNA was then extracted using the Power Viral Environmental RNA/DNA Isolation Kit (Qiagen, Hilden, Germany). The DNA was fragmented into 350 bp pieces using a Covaris M220 ultrasonicator (Covaris, Woburn, MA, United States), and libraries were constructed using the Accel-NGS 1S Plus DNA Library Kit (Integrated DNA Technologies (IDT), Ann Arbor, Michigan, United States). High-throughput sequencing was conducted on an Illumina HiSeq 2500 platform.

### 2.6 Metavirome data processing

The raw data obtained from the Illumina HiSeq platform were processed using Fastp software for quality trimming, retaining high-quality reads to obtain clean reads (Chen et al., 2018). The clean reads were then assembled into contigs using the assembly software metaSPAdes (Nurk et al., 2017). Clean reads were subjected to a Blastx comparison against viral databases (*E*-value < 1e-5) to obtain species classification annotations (Buchfink et al., 2015). The viral database integrated the NCBI non-redundant (NR) database, Refseq virus database, and the bacteriophage database from the PHAST website. The viral sequences mapped to the databases were further analyzed for open reading frame (ORF) prediction using Prodigal v2.6.3 with default parameters (Hyatt et al., 2010). The predicted protein sequences were then compared with the KEGG protein database (*E*-value < 1e-5), and viral functions were categorized using MEGAN 6 (Huson et al., 2007). The viral contigs were also annotated for CAZyme auxiliary metabolic genes using the dbCAN meta server online tool (Zhang et al., 2018).

### 2.7 Statistical analysis

Statistical analyses were performed using SPSS, including one-way analysis of variance (ANOVA) and homogeneity of variance tests. Abundance bar plots were generated using Origin software. R v4.4.1 was used as the main plotting tool to create correlation heatmaps and random forest models to explore the environmental drivers of viral and microbial abundance. Relative abundance plots were also created, and correlation analyses were conducted to reveal the relationship between viral community composition and environmental drivers. Functional prediction plots and relative abundance plots of auxiliary carbohydrate-active enzyme genes were generated to investigate the potential roles of viruses.

## 3 Results

### 3.1 Soil physicochemical properties across forest succession

The soil pH of secondary forests at different successional stages is acidic and shows a trend of increasing initially and then decreasing. As the forest age increased, significant differences were observed in SWC, NH_4_^+^-N, NO_3_^−^-N, TN, and TC. SWC continuously increased, with the 40-year and 100-year-old secondary forest soils showing significantly higher water content than the other forest stands. NH_4_^+^-N and NO_3_^−^-N exhibited opposite trends in the 40-year-old secondary forest soil, with NH_4_^+^-N reaching its highest content and NO_3_^−^-N reaching its lowest. TN and TC contents significantly increased, reaching their maximum in the 100-year-old secondary forest soil (Supplementary Table S1).

### 3.2 Virus abundance and environmental drivers

**Figure 1:**
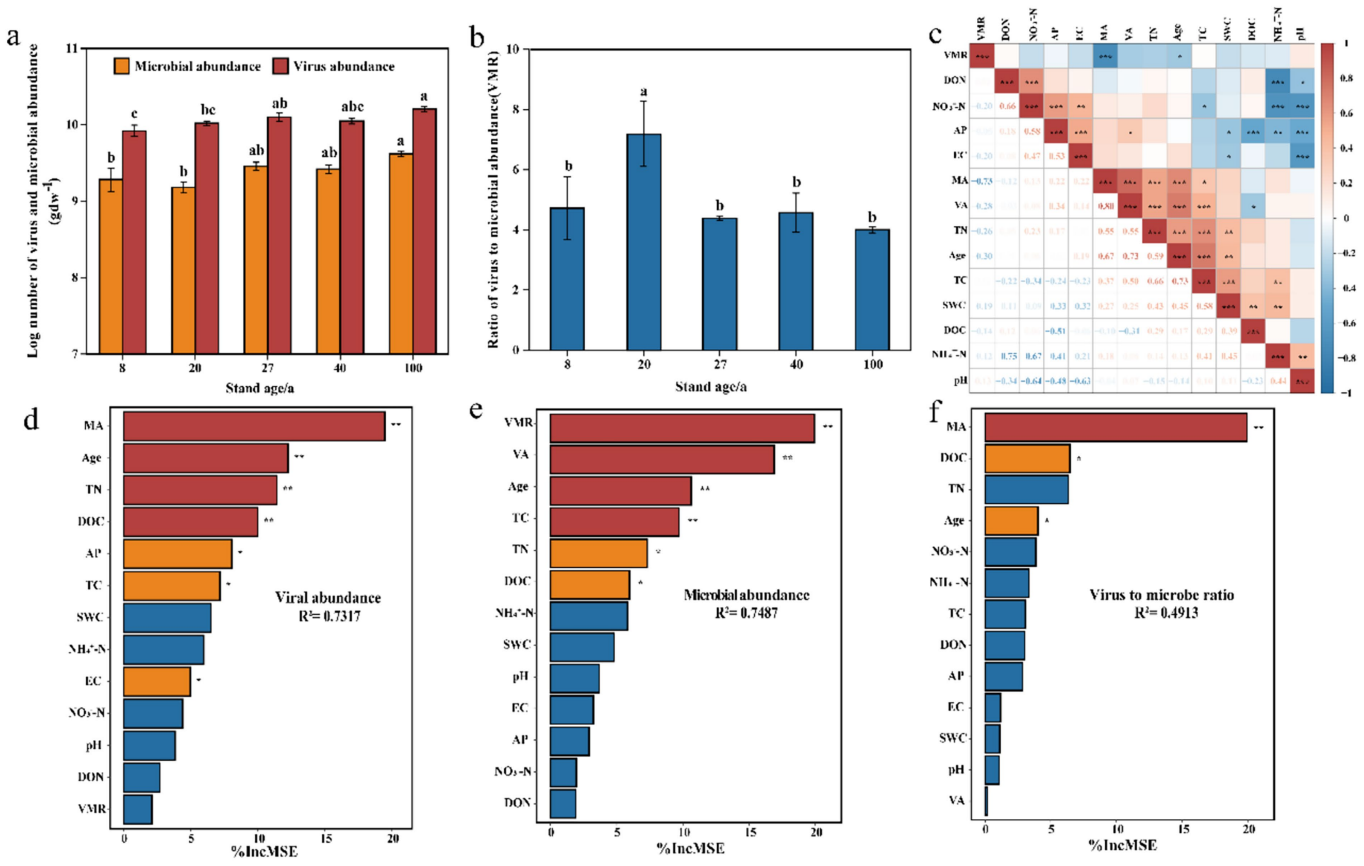
Virus abundance, microbial abundance, VMR and their environmental drivers. **(a,b)** Comparison of soil virus abundance, microbial abundance, and VMR across different forest ages in secondary forests. **(c)** Correlation between virus abundance, microbial abundance, VMR, and soil physicochemical properties. **(d–f)** Random forest model assessing the relative importance of factors influencing virus and microbial abundance, and VMR. Asterisks indicate significant effects (*p < 0.05, **p < 0.01).

The abundance of viruses and microorganisms in the soil was directly counted using fluorescence microscopy. The abundance of virus-like particles (VLPs) ranged from 8.58 × 10^9^ gdw^−1^ to 1.63 × 10^10^ gdw^−1^, while microbial abundance ranged from 1.56 × 10^9^ gdw^−1^ to 4.18 × 10^9^ gdw^−1^. The virus-to-microbe ratio (VMR) in the soil ranged from 4.0 to 7.2. With increasing forest age, both soil VLP abundance and microbial abundance gradually increased, reaching their maximum values in the 100-year-old secondary forest soils (Figure 1a). The VMR of the 20-year-old secondary forest was significantly higher than that of the other forest stands (Figure 1b). Combining the two figures, it is apparent that as microbial abundance increases, the VMR decreases.

Pearson correlation analysis was performed to assess the correlations between soil VLP abundance, microbial abundance, VMR, and soil physicochemical factors (Figure 1c). The results showed a significant positive correlation between VLP abundance and microbial abundance, and a significant negative correlation between VMR and microbial abundance. Forest age was significantly positively correlated with VLP abundance, microbial abundance, TC, and TN, while it was negatively correlated with VMR. Subsequently, a random forest model was used to assess the relative importance of abiotic factors (SWC, TC, TN, AP, NH_4_^+^-N, NO_3_^−^-N, DOC, DON, pH, EC), biological factors (VLP and microbial abundance, VMR), and forest stand factors (Age) on VLP and microbial abundance, as well as VMR. The results indicated that microbial abundance was the most important factor affecting virus abundance and VMR, while VMR and virus abundance were the main factors influencing microbial abundance. Forest age ranked just after these three biological factors in terms of importance and played a significant role (Figures 1d–f).

### 3.3 Viral and bacterial community diversity

**Figure 2:**
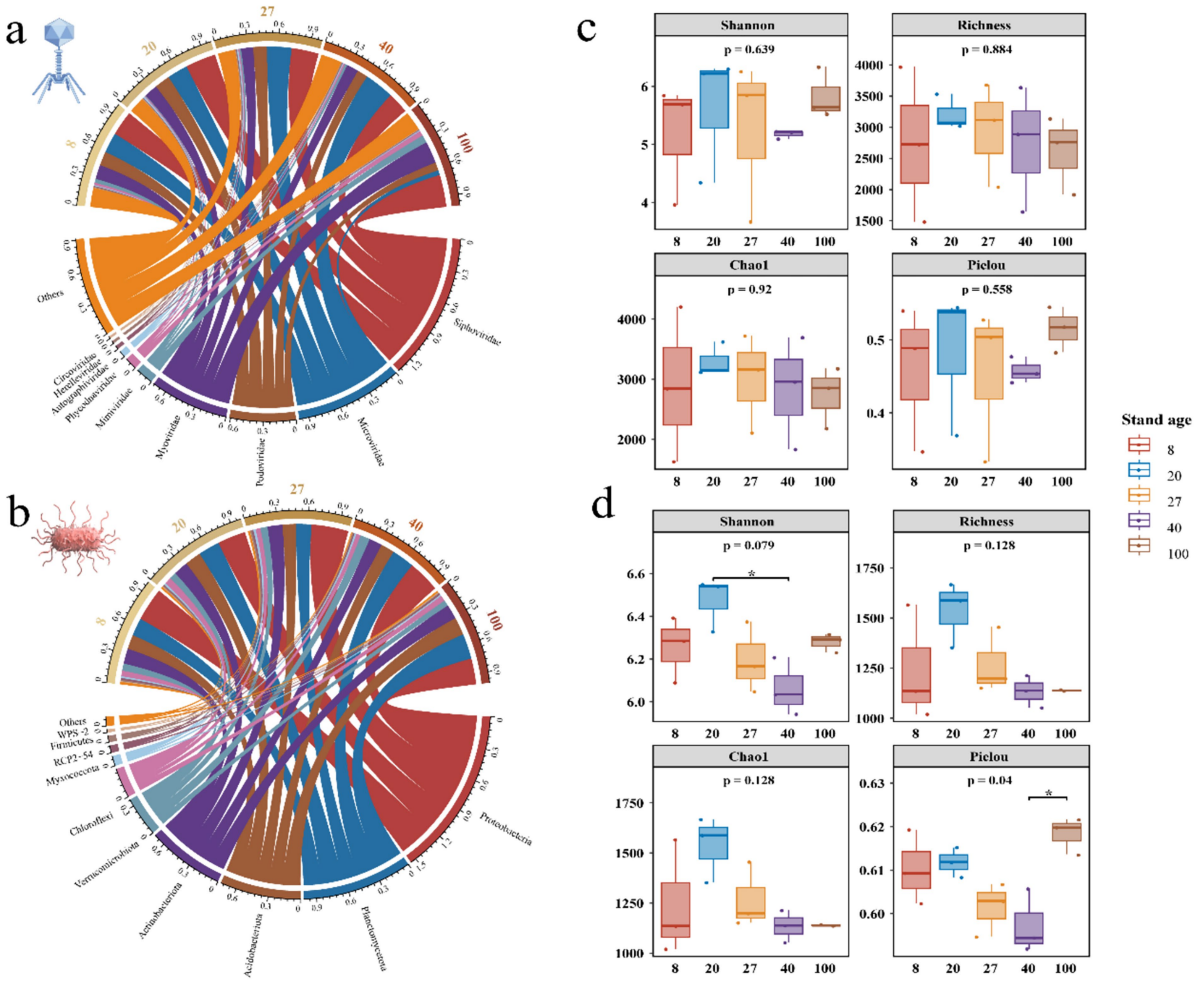
Comparison of viral and bacterial community composition, richness, evenness and their influencing factors. **(a)** Composition of viral communities at the family level across different forest ages in secondary forest soils. **(b)** Composition of bacterial communities at the phylum level. **(c,d)** Comparison of viral and bacterial richness and evenness across different forest ages.

A total of 30 viral orders, 61 viral families, and 671 viral genera were identified from the viral metagenomic data of soil samples. This study found that the viral community was predominantly composed of tailed bacteriophages, which infected bacteria, accounting for 55.77% of the community. At the family level, Siphoviridae had the highest average relative abundance, followed by Microviridae, Podoviridae, and Myoviridae (Figure 2a). Bacterial annotation results showed that, in 15 secondary forest soil samples, bacteria spanned across 20 bacterial phyla, including Proteobacteria, Planctomycetota, Acidobacteria, and others (Figure 2b). Among them, Proteobacteria dominated, accounting for 31.13% of the total bacterial population, followed by Planctomycetota, Acidobacteria, and Actinobacteria.

During the succession of secondary forests, the composition proportions of viral communities underwent significant changes, with the 100-year-old forest representing a notable turning point. Myoviridae, Siphoviridae, Mimiviridae, and Phycodnaviridae exhibited increasing trends, reaching their highest relative abundance in the 100-year-old soil. Conversely, Podoviridae and Microviridae exhibited the opposite trend, showing the lowest relative abundance in the 100-year-old soil. Microviridae significantly increased in the 40-year-old soil, but sharply decreased in the 100-year-old soil (Supplementary Figure S1a). The bacterial community composition showed slight fluctuations in relative abundance across different forest ages, but no drastic changes occurred in the dominant bacterial phyla, suggesting that soil microorganisms may possess a certain degree of ecological stability (Supplementary Figure S1b). There was no significant difference in viral community diversity across different forest ages (Figure 2c), while bacterial community evenness showed significant differences, indicating that forest age may affect bacterial community evenness and distribution patterns, especially at higher forest ages (Figure 2d).

**Figure 3:**
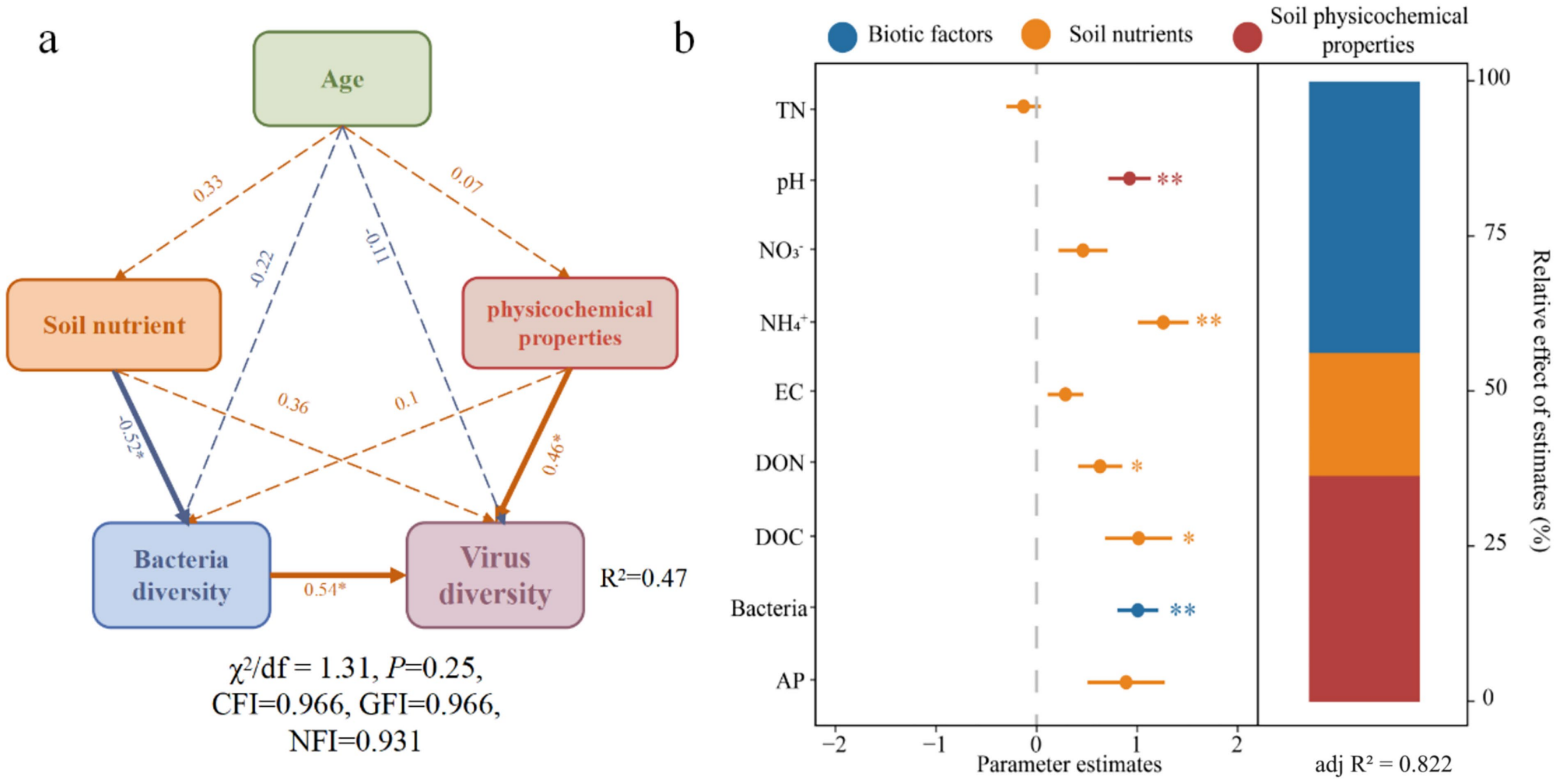
Factors influencing viral community diversity and their relative contribution. **(a)** The segmented structural equation model integrated the direct and indirect effects of forest age, soil nutrients, soil physicochemical properties, and bacterial diversity on viral diversity. The orange and blue arrows represent positive and negative influences, respectively. The numbers beside the arrows are the path coefficients of the partial regression, showing the standardized effect sizes. Dashed lines indicate no effect, while solid lines represent significant effects. Age refers to forest age indicators; Soil nutrient refers to TN, NO_3_^−^, NH_4_^+^, DON, DOC, AP indicators; Physicochemical properties refers to pH and EC indicators; bacterial and viral diversity is represented by the Richness indicator. All components were first normalized within the range of 0 to 1 according to the maximum-minimum value, with the average of the standardized scores for Soil nutrient and physicochemical properties. **(b)** The relative contribution of different predictive factors. The circles display the average parameter estimates (standardized regression coefficients) for model predictive factors associated with the 95% CI. The relative importance of each factor is represented as the percentage of variance explained. Asterisks indicate significant effects (*p < 0.05, **p < 0.01). Biotic factors represent bacterial diversity; Soil nutrients include TN, NO_3_^−^, NH_4_^+^, DON, DOC, AP; Physicochemical properties represent pH, EC.

The structural equation model (SEM) indicated that soil nutrients had a significant negative effect on bacterial diversity, while soil physicochemical properties and bacterial diversity positively and significantly influenced viral diversity (Figure 3a). Specifically, bacterial diversity and soil physicochemical properties directly influenced viral diversity, whereas soil nutrients indirectly affected viral diversity by influencing bacterial diversity (Supplementary Figure S2). Next, a mixed linear model was used to explore the specific factors affecting viral community diversity. The results revealed that bacterial community diversity, soil pH, and NH_4_^+^-N all had a highly significant positive impact on viral diversity, with soluble organic carbon and organic nitrogen having a slightly lesser effect. Among these factors, bacterial community diversity contributed the most, followed by soil pH, while the contribution of soil nutrients was relatively small (Figure 3b).

The neutral community model showed that both viral and bacterial community construction were mainly driven by neutral processes (i.e., the balance between diffusion and drift). However, the environmental selection of the bacterial community played a stronger role than that of the viral community. At the same time, both communities had high migration rates, with the migration rate of viruses being significantly higher than that of bacteria. This could be due to viruses depending on host diffusion, whereas the diffusion of host bacteria is more limited (Supplementary Figure S3).

### 3.4 Viral-mediated carbon cycling potential and environmental drivers

**Figure 4:**
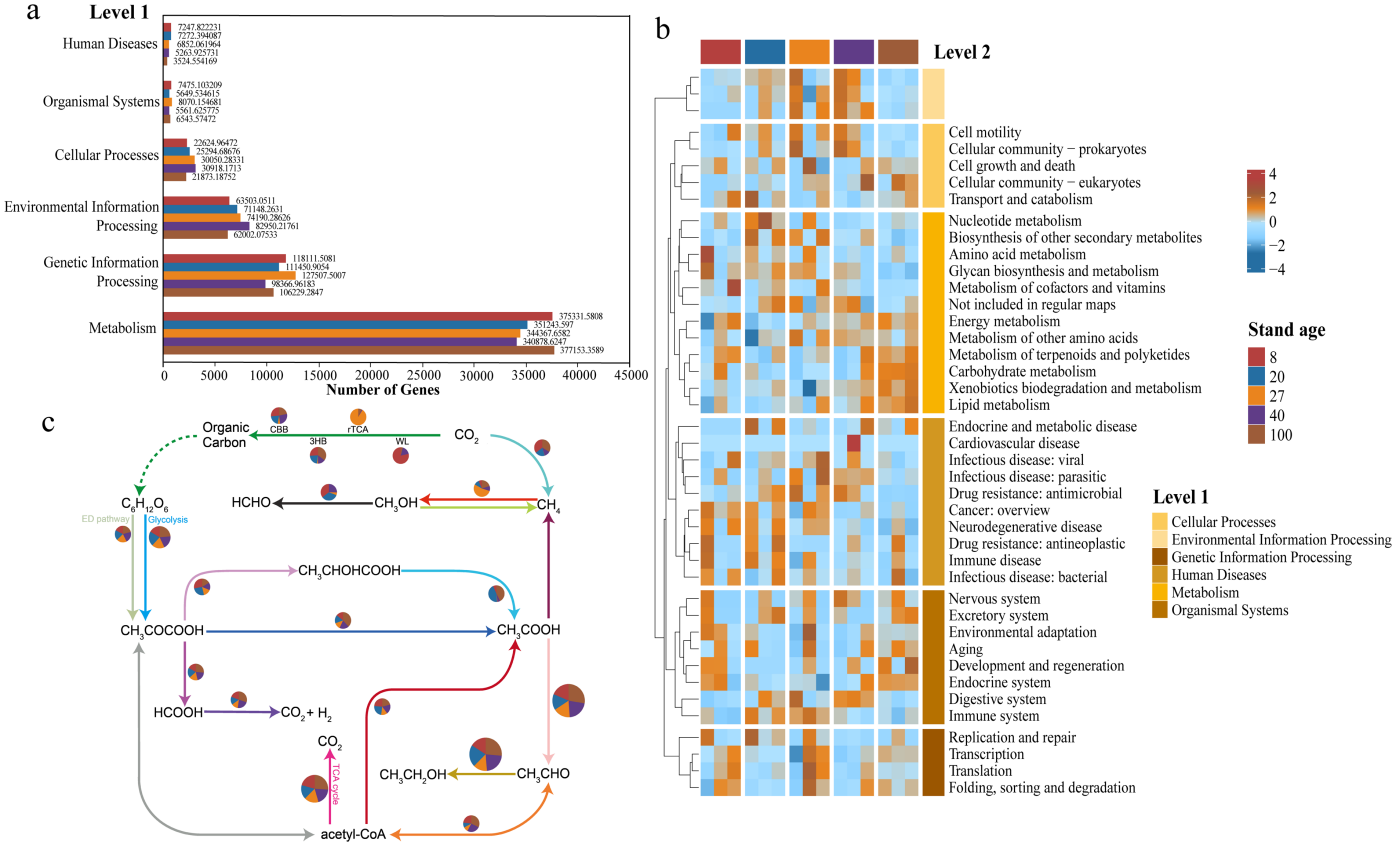
Viral functional characteristics. **(a)** Analysis of the number of functional genes of soil viruses in subtropical secondary forests at different forest ages based on the KEGG database at the Level 1 functional classification. **(b)** Functional prediction of soil viruses in subtropical secondary forests at Level 2. **(c)** Analysis of the relative abundance of carbon cycling functional genes involved in carbon cycling pathways across different forest age samples using the DiTing database. The size of the pie charts represents the total relative abundance of each pathway. CBB, Calvin-Benson-Bassham cycle; rTCA, reductive citric acid cycle; WL, Wood-Ljungdahl pathway; 3HB, 3-hydroxypropionate bicycle; DHc, dicarboxylate-hydroxybutyrate cycle.

The obtained KO numbers were compared with the KEGG Orthology database to categorize the functional annotations of the secondary forest soil virome. At Level 1, six viral functions were annotated, with the metabolic function Metabolism comprising the largest proportion, accounting for 67.1%, while Human Diseases related to human disease functions had the smallest proportion, at only 1.04% (Figure 4a). At Level 2, a further analysis of metabolic function genes revealed that carbohydrate metabolism genes had the highest relative abundance. Amino acid metabolism, energy metabolism, and cofactor and vitamin metabolism genes also represented substantial proportions. Cluster analysis using abundance heatmaps showed that as forest age increased, the relative abundance of carbohydrate metabolism genes, which were the most abundant, also increased (Figure 4b). Redundancy analysis (RDA) revealed that environmental factors collectively explained 50.37% of the total variation in soil viral functional profiles. Significant differences in viral functions were observed among different forest ages, and shifts in viral community functions were significantly correlated with soil pH (Supplementary Figure S4).

Based on functional annotation of KO abundance and metabolic pathway reconstruction using DiTing software, it is inferred that the functional potential of soil viromes in carbon cycling pathways may differ significantly across different successional stages of secondary forests (Figure 4c). In young forests, viral functional genes are primarily enriched in carbon fixation pathways, particularly the CBB cycle and the Wood-Ljungdahl (WL) pathway, which may suggest that viruses participate in the carbon fixation process by regulating autotrophic microorganisms. As forest age increases, the functions of the mid-aged forest virome gradually shift toward respiration and decomposition-related metabolic pathways, indicating enhanced heterotrophic processes. In the near-mature forest stage, viral-associated genes are significantly enriched in the reductive TCA cycle (rTCA), suggesting that the hosts at this stage may possess stronger carbon fixation potential. In mature and overmature forests, the viral functional potential is more concentrated in acetate metabolism and carbon release-related pathways, which may indicate that the soil viral community promotes organic matter decomposition and carbon release through host regulatory mechanisms.

**Figure 5:**
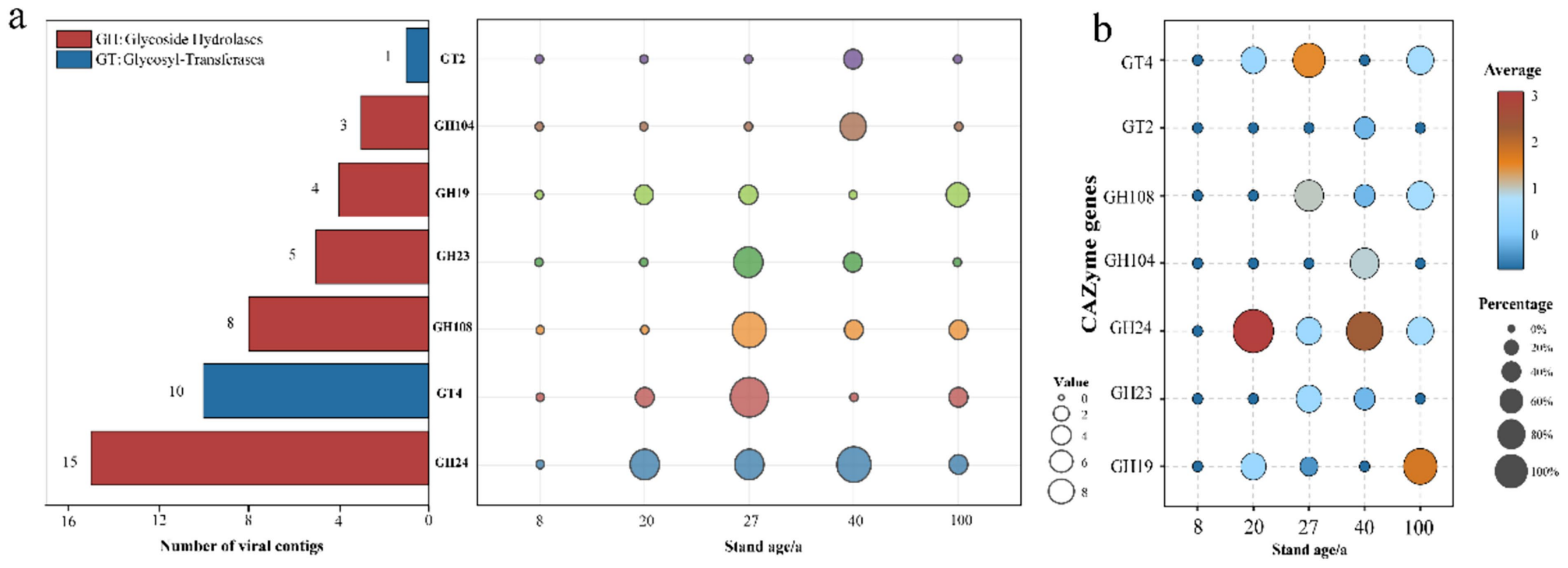
Abundant auxiliary CAZyme in subtropical secondary forest. **(a)** Annotation of viral carbohydrate metabolism-related contigs in the CAZy database. **(b)** Relative abundance of viral auxiliary carbohydrate-active enzyme genes in each sample.

To further elucidate the role of viruses in the soil carbon cycle, we utilized the dbCAN server to annotate carbohydrate-active enzymes (CAZymes) based on the identification of CAZymes characteristic domains for 3,278 vOTUs. The results showed that 46 types of viral CAZymes were present in the secondary forest soils, classified into glycoside hydrolases (GH) and glycosyltransferases (GT), with GH24 and GT4 representing the highest proportions. No auxiliary carbohydrate metabolism genes were detected in the 8-year-old secondary forest soil, while the 27-year-old soil had the most detected metabolism genes. GT2 and GH104 genes were only found in the 40-year-old soil. The number of metabolism genes showed an increasing-decreasing trend with increasing forest age (Figure 5a). According to the relative abundance chart, the most abundant genes changed with increasing forest age. In the 20-year-old and 40-year-old soils, GH24 was the most abundant gene, while GT4 dominated in the 27-year-old soil. In the 100-year-old soils, GH19 occupied the highest proportion (Figure 5b).

## 4 Discussion

### 4.1 Major drivers of soil viral communities and functions in subtropical secondary forests

Previous studies have shown that the abundance of viruses in soil is influenced by various factors, including soil type, physicochemical properties, and other environmental conditions (Liang et al., 2021). Correlation analysis and random forest models revealed that the abundance of soil viruses is highly significantly positively correlated with forest age, host microbial abundance, TN, and TC, indicating that forest age is a key environmental factor driving changes in soil virus abundance. Microbial abundance is a crucial biological factor affecting soil virus abundance, while TN and TC are essential physicochemical factors influencing soil virus abundance.

The structural equation model indicates that bacterial diversity has a direct and positive impact on viral diversity, followed by soil physicochemical properties, while soil nutrients indirectly influence viral diversity by affecting bacterial diversity. The mixed linear model and structural equation model mutually validated this finding, revealing that bacterial community diversity significantly influenced viral community diversity, with soil pH having the second most significant impact, and soil nutrients contributing relatively less. Studies on soybean rhizosphere microbiomes have shown that the dynamic diversity of viral communities is closely related to the succession of host bacteria. During the flowering period, the host range of viruses expands and infects specific functional groups, suggesting that host diversity drives viral infection specificity and functional differentiation (Cheng et al., 2024). In this study, the abundance of host microorganisms was found to be a key biotic factor influencing soil viral abundance, further confirming the significant impact of soil bacteria on viral communities. Consistent with numerous previous studies, soil pH was also identified in this study as a key environmental factor influencing viral community diversity (Lee et al., 2022; Ma et al., 2024; Gu et al., 2025). The adsorption of viruses onto solid surfaces is a common phenomenon, and this process is closely related to the electrostatic properties of soil particle surfaces, which are largely governed by soil pH (Kapuscinski and Mitchell, 1980). Research on agricultural red soils has shown that soil pH can affect the adsorption of viruses onto soil particles, with viral adsorption decreasing as soil pH increases, thereby affecting viral reproduction (Chen et al., 2014; Bi et al., 2021), suggesting that pH directly influences viral diversity by affecting virus adsorption. Moreover, this study identified pH as a major environmental factor influencing viral functional potential. This may be attributed, on one hand, to the critical role of pH in regulating host growth and metabolism, which in turn indirectly affects viral functions. On the other hand, pH fluctuations may directly influence the expression and functionality of AMGs (Jansson and Wu, 2023). Studies indicate that soil pH modulates viral community composition, thereby regulating the capacity of viruses to carry AMGs. Distinct viral assemblages harbor differentiated AMG repertoires, with concomitant variations in functional expression (Chen et al., 2025).

### 4.2 The impact of temporal succession on soil viral communities in subtropical secondary forests

In this study, the viral abundance in secondary forest soils increased with forest age and reached its maximum in the 100-year-old soil samples. Additionally, the abundance of host microorganisms and the contents of TN and TC also peaked in the 100-year-old forest soils. The positive effect of stand age on viral communities may be attributed to higher levels of carbon and nitrogen nutrients in older forest soils, as indicated by physicochemical analyses. These enriched conditions likely support a more abundant and diverse host microbial community, thereby providing favorable environments for viral replication and increasing opportunities for infection (Srinivasiah et al., 2008).

Based on the virus species annotation results, the dominant viral groups in secondary forest soils were tail-containing bacteriophages, with Siphoviridae having the highest average relative abundance, consistent with previous studies (Adriaenssens et al., 2017; Zheng et al., 2021). During the development of secondary forest stands, the relative abundance of various viral communities showed significant changes, with Myoviridae, Siphoviridae, Mimiviridae, and Phycodnaviridae increasing fluctuatingly, while Podoviridae and Microviridae decreased fluctuatingly, reaching their highest and lowest relative abundances, respectively, in the 100-year-old forest soils. At the same time, Microviridae significantly increased in the 40-year-old forest soils but sharply decreased in the 100-year-old soils. However, the differences in the richness and evenness of viral communities among soils of different forest ages were not significant, which may be because in secondary forests with high host density, lysogenic bacteriophages tend to integrate into the host genome, thus reducing community fluctuations. This phenomenon aligns with the classic Piggyback-the-Winner (PtW) model (Dion et al., 2020; Correa et al., 2021). According to the PtW model, at high host densities, phages tend to adopt the lysogenic cycle, integrating into the host genome, and the virus-to-host ratio is suppressed (Knowles et al., 2016). In this study, the higher the microbial abundance in subtropical secondary forests, the lower the VMR, indicating that the distribution of viruses follows the PtW model. The neutral community model also suggests that the distribution of viral communities in secondary forest soils is primarily governed by neutral processes (diffusion-drift balance), with high migration rates and moderate goodness-of-fit jointly supporting the phenomenon of no significant differences in viral community richness and evenness among different forest ages (Jia et al., 2025).

### 4.3 Potential impact of soil viruses on carbon cycling in subtropical secondary forests

Relevant studies have shown that viruses can indirectly regulate host community life processes through their encoded auxiliary metabolic genes in an upward regulatory manner, thereby influencing the soil carbon cycle (Hurwitz and U'Ren, 2016; Wang L. et al., 2024). In this experiment, KEGG functional annotation predicted that in the soil virome of subtropical secondary forests across different forest ages, there are numerous functional genes related to carbohydrate metabolism. These genes help hosts decompose and utilize complex carbohydrates, thereby enhancing the environmental adaptability of viruses and hosts (Middelboe and Brussaard, 2017). This suggests that after viral infection of host microorganisms, viruses may influence host microbes through the expression of carbon metabolism-related genes, indirectly participating in the forest soil carbon cycling process. Subsequently, the carbon cycling pathways of the soil virome were predicted, revealing that the functional potential of soil viruses in different forest ages showed clear successional changes, reflecting an ecological functional transition from carbon fixation to organic matter decomposition and then to carbon release. This indicates that viruses play a dynamic functional role during forest succession, and the metabolic functional genes they carry may influence the fixation, transformation, and release of carbon by host microbes, thereby affecting the forest soil carbon cycle.

Therefore, to further analyze the potential role of auxiliary metabolic genes in the carbon cycle within soil viruses, we annotated the CAZymes in the soil virome genomes. The results confirmed the presence of a rich diversity of CAZymes in the subtropical secondary forest soil virome, with the majority being GH and a smaller portion being GT. GH enzymes can promote the degradation of complex carbohydrates, thereby increasing energy production and enhancing host metabolism (Anderson et al., 2017), while GT enzymes catalyze the attachment of activated sugars to various receptor molecules within the organism, which is associated with the synthesis and transfer of carbohydrates (Ban et al., 2012).

Based on the integration of carbon metabolic pathway analysis and CAZyme (carbohydrate-active enzyme) annotations, a coordinated pattern was observed, jointly highlighting the potential role of soil viruses in the carbon cycling processes of subtropical secondary forests. In the early successional stage, forest soils were primarily characterized by carbon fixation and storage, with relatively weak microbial decomposition activity (Wang Y. et al., 2024); accordingly, no CAZyme genes were detected in the viral metagenomes. At the mid-successional stage, carbon decomposition and respiration became more prominent, and CAZyme genes belonging to the glycoside hydrolase (GH) and glycosyltransferase (GT) families began to accumulate, suggesting that viruses may regulate host carbon metabolism, particularly in terms of carbon source degradation and transformation. The late-mid successional stage appeared to be a critical phase in the soil carbon cycle, during which viral functions shifted toward a more balanced profile of carbon assimilation and release. The increasing abundance of GH and GT genes in this phase indicated that viruses not only participated in carbon fixation but also actively promoted the breakdown and turnover of carbon substrates. In the mature forest stage, viral functional potential further transitioned toward carbon mobilization and release. Organic matter degradation and carbon release peaked during this period, with GH and GT gene abundances remaining high. This suggests that viruses may promote carbohydrate degradation, glycosyl transfer, and resynthesis by modulating host metabolic processes, thereby actively contributing to carbon cycling. Despite ecosystem aging and reduced carbon sequestration capacity in the old-growth stage, a substantial amount of organic matter still required decomposition. Microbial activity continued to accelerate carbon release, and viral CAZyme genes maintained moderate abundance, facilitating the return of carbon to the atmosphere. Collectively, these findings indicate that soil viruses play a potentially important and dynamic role in regulating carbon cycling across different successional stages in subtropical secondary forest soils.

## 5 Conclusion

Our study provides experimental evidence that soil viral communities in subtropical secondary forests exhibit distinct distribution patterns across different stand ages. Viral abundance increased progressively with forest age, and the distribution of viral communities conformed to the power-law with exponential cutoff (PtW) model. Bacterial diversity and soil pH were identified as key drivers shaping viral community diversity. In addition, this study confirms the potential involvement of soil viruses in carbon cycling during forest development. Functional potential analysis revealed a successional shift from carbon assimilation toward carbon release, highlighting the dynamic ecological role of viruses in mediating soil carbon turnover. However, transcriptional validation of viral functional genes was not addressed in this study. Future research should incorporate RT-qPCR or viral metatranscriptomics to further elucidate how viral metabolic genes are expressed and functionally engaged in key carbon cycling pathways. Overall, this research provides temporal insights into the distributional dynamics of soil viruses during subtropical forest succession, expanding our understanding of the interactions among secondary forest development, soil viral ecology, and carbon cycling processes.

## Data Availability

The data presented in the study are deposited in the NCBI, accession number PRJNA1310042. This data can be found here: https://www.ncbi.nlm.nih.gov/bioproject/1310042.
