# Supplementary material for: Soil viruses drive carbon turnover during subtropical secondary forest succession
Source: Front Microbiol. 2025 Sep 19;16:1633379. doi: 10.3389/fmicb.2025.1633379 (PMC12491279; doi:10.3389/fmicb.2025.1633379)
Supplement: Supplementary file 1 [file Table_1.docx]

Soil Viruses Drive Carbon Turnover During Subtropical Secondary Forest Succession

Xingyi Chen^a,b^, Danting Yu^a,b^*, Yuting Yan^a,b^, Chengyu Yuan^a,b^, Jizheng He^a,c^

^a^ *College of Geographic Sciences, Fujian Normal University, Fuzhou, Fujian 350117, China*

^b^ *Fujian Provincial Key Laboratory for Subtropical Resources and Environment, Fujian Normal University, Fuzhou 350117, China*

^c^ *School of Agriculture, Food and Ecosystem Sciences, Faculty of Science, The University of Melbourne, VIC 3010, Australia*

***Correspondence**

Dr. Danting Yu

Tel: +86 591 83465214

E-mail: dty@fjnu.edu.cn

***Supplementary Information***

**Supplementary Tables**

Table. 1 Physical and chemical properties of natural forest soil under different forest ages

| **Age(year)** | **8** | **20** | **27** | **40** | **100** |
| --- | --- | --- | --- | --- | --- |
| **pH** | 4.48±0.30c | 4.77±0.13a | 4.70±0.04ab | 4.73±0.13a | 4.54±0.16bc |
| **EC (μS/cm)** | 68.89±6.01a | 53.33±12.25b | 65.56±13.33a | 57.78±9.72ab | 70.00±15.81a |
| **SWC(%)** | 33.10±5.05b | 32.47±6.05b | 34.32±2.42b | 41.78±4.04a | 40.51±7.05a |
| **NH_4_ ^+^-N (mg/kg)** | 13.17±7.87ab | 8.69±2.32b | 11.55±0.62ab | 16.38±6.22a | 13.66±10.71ab |
| **NO_3_ ^-^-N (mg/kg)** | 5.80±6.00a | 2.54±2.38ab | 3.27±3.45ab | 1.23±0.45b | 5.04±6.14ab |
| **DON（mg/kg）** | 11.07±6.01a | 13.45±4.58a | 12.95±5.66a | 9.06±4.41a | 12.3±4.17a |
| **DOC（mg/kg）** | 353.41±71.34a | 305.18±95.72a | 311.56±30.91a | 361.48±21.52a | 357.25±35.26a |
| **TN（g/kg）** | 2.02±0.14b | 1.70±0.15c | 1.90±0.14bc | 1.98±0.26b | 2.42±0.44a |
| **TC（g/kg）** | 26.00±4.42b | 28.13±3.76b | 25.61±2.56b | 29.94±3.19b | 38.38±6.32a |
| **AP（mg/kg）** | 3.61±0.67a | 3.21±0.96a | 3.49±0.99a | 3.08±0.26a | 3.44±0.62a |

The results are presented as means±standard deviation. One-way ANOVA with Duncan's test was used to determine statistical differences between groups, with appropriate labeling (i.e., a, b, c) for each group. Different lowercase letters indicate significant differences between forest ages (*P* < 0.05). The same applies to other comparisons.

**Supplementary Figures**


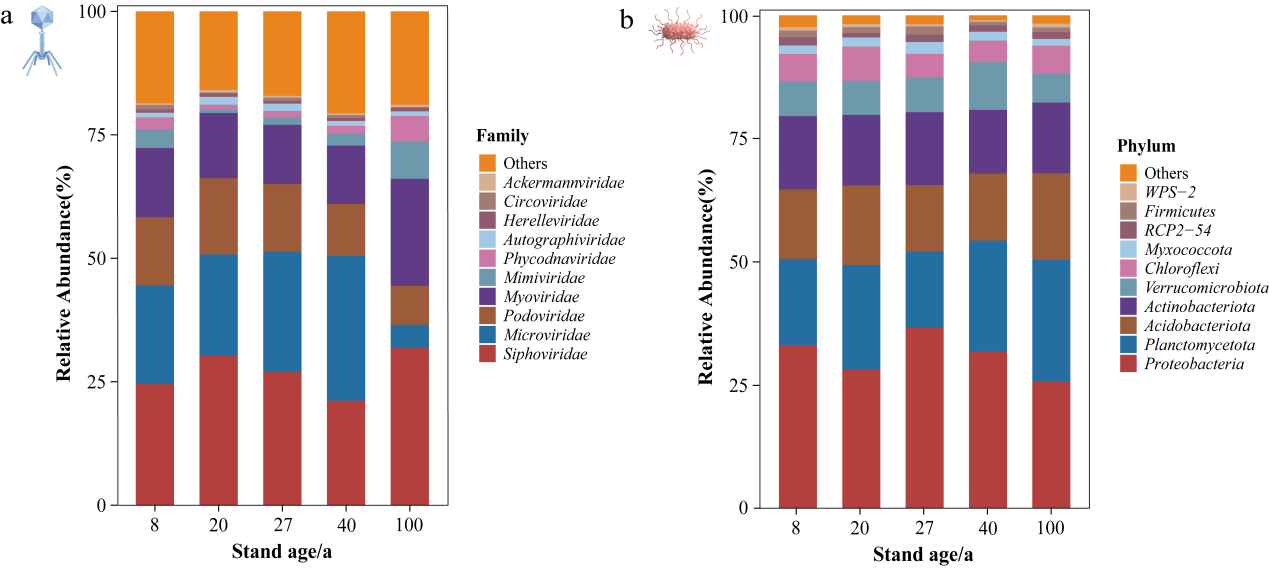


Fig. 1Changes in the species composition of viruses at the family level. (a) and bacteria at the phylum level. (b) in soils from forests of different ages.


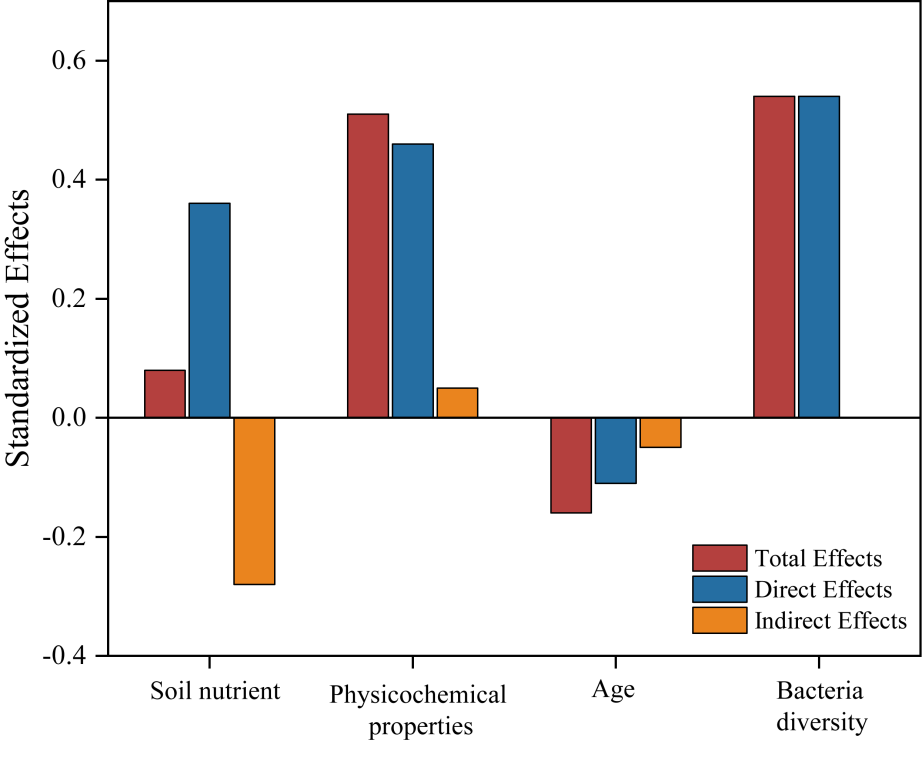


Fig. 2 Direct and indirect effects of different factors on viral diversity.


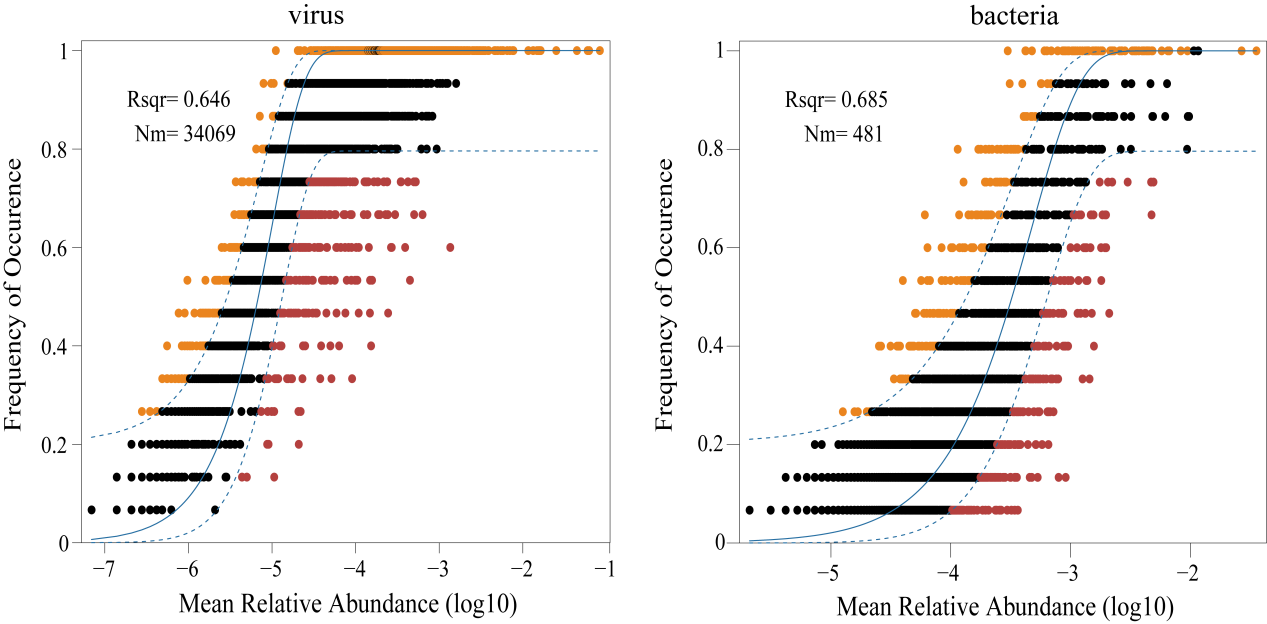


Fig. 3 Neutral community model of viruses and bacteria.


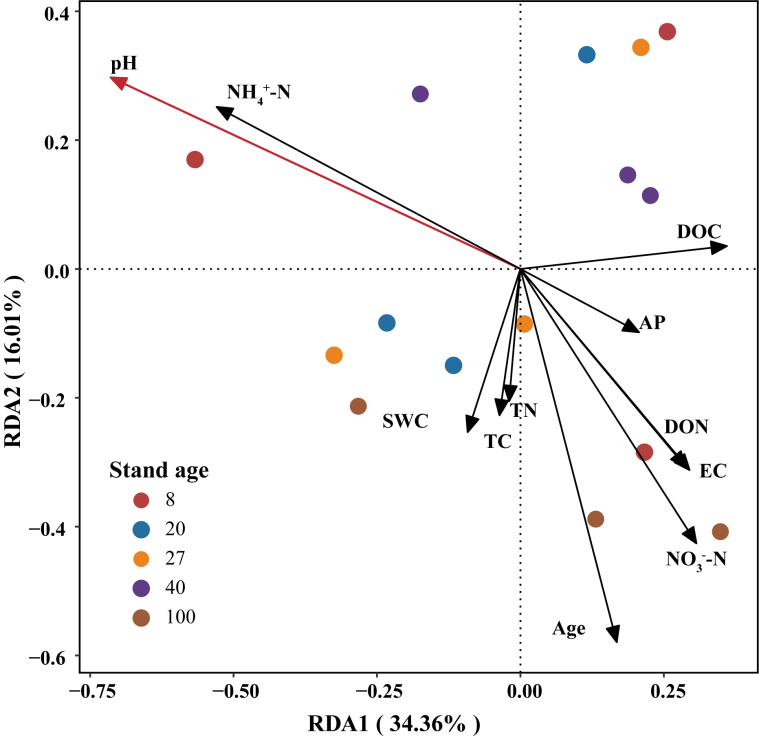


Fig. 4 Redundancy analysis of environmental drivers of soil viral functions in secondary forests with different stand ages.
