# Supplementary figures and images for: Soil viruses drive carbon turnover during subtropical secondary forest succession
Source: Front Microbiol. 2025 Sep 19;16:1633379. doi: 10.3389/fmicb.2025.1633379 (PMC12491279; doi:10.3389/fmicb.2025.1633379)

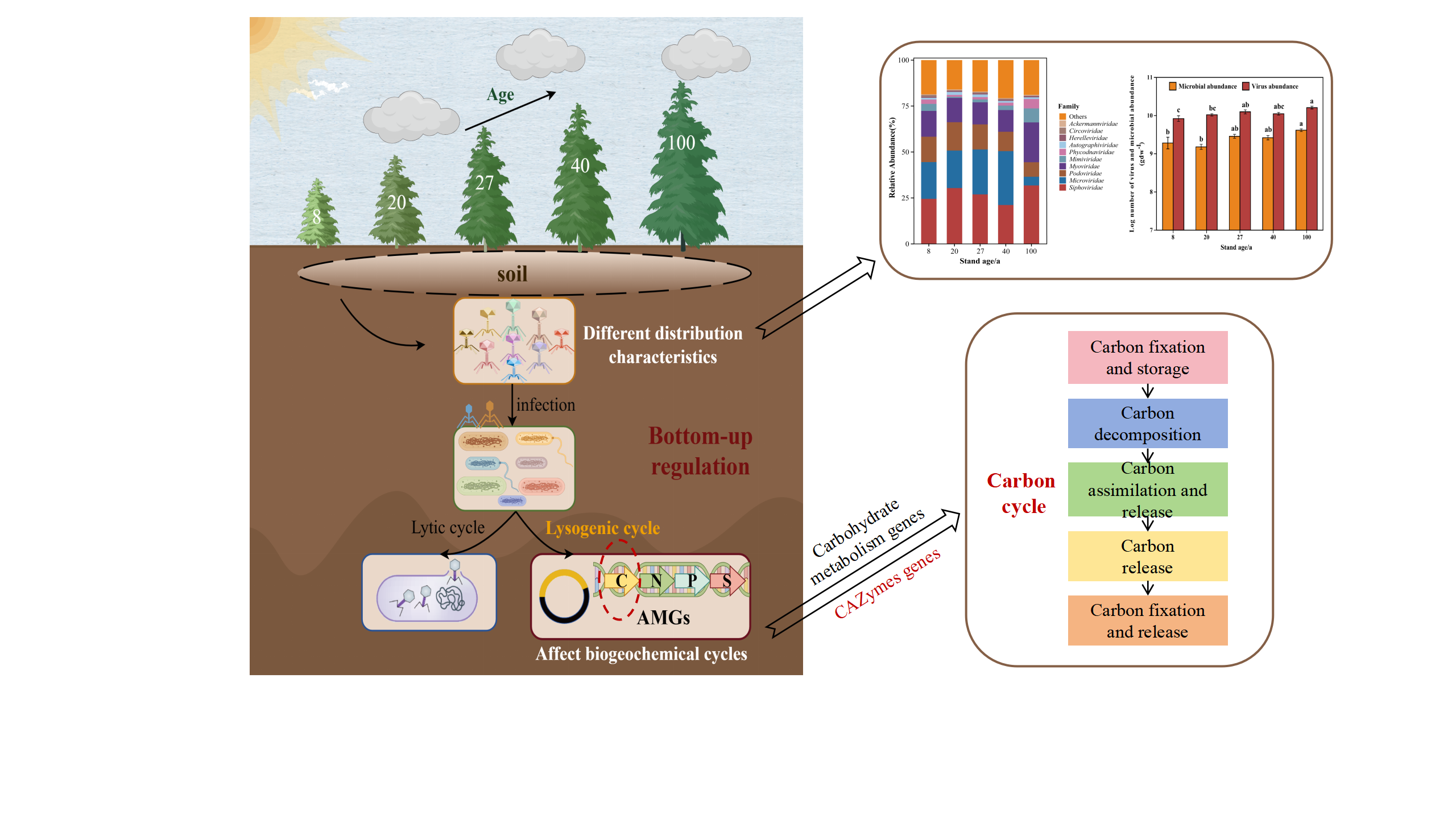

Supplement: Supplementary file 2 [file Image_1.TIF]
